# A Phase 1 study of intravenous infusions of tigecycline in patients with acute myeloid leukemia

**DOI:** 10.1002/cam4.845

**Published:** 2016-10-13

**Authors:** Gregory A. Reed, Gary J. Schiller, Suman Kambhampati, Martin S. Tallman, Dan Douer, Mark D. Minden, Karen W. Yee, Vikas Gupta, Joseph Brandwein, Yulia Jitkova, Marcela Gronda, Rose Hurren, Aisha Shamas‐Din, Andre C. Schuh, Aaron D. Schimmer

**Affiliations:** ^1^ University of Kansas Cancer CenterKansas CityKansas; ^2^ David Geffen School of Medicine at UCLALos AngelesCalifornia; ^3^ Leukemia ServiceDepartment of MedicineOncology and Pathogenesis ProgramMemorial Sloan Kettering Cancer CenterNew YorkNew York; ^4^ Princess Margaret Cancer CentreUniversity Health NetworkTorontoOntarioCanada; ^5^ Department of MedicineUniversity of AlbertaEdmontonAlbertaCanada

**Keywords:** Cox‐1, Cox‐4, mitochondrial protein synthesis, pharmacodynamics, pharmacokinetics

## Abstract

Acute myeloid leukemia (AML) cells meet the higher energy, metabolic, and signaling demands of the cell by increasing mitochondrial biogenesis and mitochondrial protein translation. Blocking mitochondrial protein synthesis through genetic and chemical approaches kills human AML cells at all stages of development in vitro and in vivo. Tigecycline is an antimicrobial that we found inhibits mitochondrial protein synthesis in AML cells. Therefore, we conducted a phase 1 dose‐escalation study of tigecycline administered intravenously daily 5 of 7 days for 2 weeks to patients with AML. A total of 27 adult patients with relapsed and refractory AML were enrolled in this study with 42 cycles being administered over seven dose levels (50–350 mg/day). Two patients experienced DLTs related to tigecycline at the 350 mg/day level resulting in a maximal tolerated dose of tigecycline of 300 mg as a once daily infusion. Pharmacokinetic experiments showed that tigecycline had a markedly shorter half‐life in these patients than reported for noncancer patients. No significant pharmacodynamic changes or clinical responses were observed. Thus, we have defined the safety of once daily tigecycline in patients with refractory AML. Future studies should focus on schedules of the drug that permit more sustained target inhibition.

## Introduction

Relapsed and refractory acute myeloid leukemia (AML) is a highly aggressive and resistant disease that is associated with poor response to standard therapy and with poor prognosis 1. Therefore, there is an urgent need for new therapies for this disease, especially in elderly patients with limited tolerance of aggressive induction therapy.

In our previous studies, we showed that in comparison with normal hematopoietic cells, AML cells have increased reliance on oxidative metabolism, reflecting the higher energy, metabolic and signaling demands of the cell as well as reduced spare reserve capacity in the respiratory chain 2, 3, 4, 5. AML cells meet these higher demands by increasing mitochondrial protein translation carried out by mitochondrial ribosomes in the mitochondrial matrix. Mitochondrial ribosomes differ from eukaryotic cytosolic ribosomes in their structure and chemical properties 6. One key difference is that mitochondrial ribosomes use unique protein translation machinery with distinct initiation and elongation factors.

Mitochondrial elongation factor Tu (EF‐Tu) brings aminoacyl‐tRNAs in complex with GTP to the decoding site on the mitochondrial ribosome 7. Previously, we showed that an shRNA‐mediated knockdown of EF‐Tu reduced the growth and viability of AML cell lines 2. In addition, the knockdown of EF‐Tu decreased levels of Cox‐1 and Cox‐2 that are subunits of respiratory complex IV in the electron transport chain in mitochondria and are translated by mitochondrial ribosomes. However, knock down of EF‐Tu did not alter the levels of Cox‐4 that is a component of the same respiratory complex but is encoded by the nuclear genome and translated by cytoplasmic ribosomes 2, 8.

Through a chemical screen, we discovered that tigecycline kills human AML differentiated blasts as well as leukemic stem cells in vitro and in vivo by blocking mitochondrial protein synthesis 2. Tigecycline is a structural analog of tetracycline, and is mostly used as a broad‐spectrum antibiotic against gram‐positive and gram‐negative pathogens 9, 10, 11. Currently, it is approved for the treatment of adults with complicated intraabdominal, skin, soft tissue and skin structure infections, and community‐acquired bacterial pneumonia 9, 12. As an antimicrobial, tigecycline reversibly binds to the 30S subunit of the bacterial ribosome, blocking the aminoacyl‐tRNA from entering the A site, thereby inhibiting elongation of the peptide chain and protein synthesis in bacteria 13. Consistent with its design as a more potent ribosomal inhibitor than minocycline or tetracycline, tigecycline inhibits bacterial protein synthesis 3‐ and 20‐fold greater than minocycline and tetracycline, respectively 14, 15.

In AML cells, tigecycline inhibits cell growth by impairing mitochondrial protein synthesis. Similar to EF‐Tu knockdown, tigecycline decreased levels of Cox‐1 and Cox‐2, but not nuclear‐encoded Cox‐4, leading to reduced respiratory chain activity and oxygen consumption. Tigecycline killed AML blast cells and leukemic stem cells with increased mitochondrial mass and greater reliance on oxidative phosphorylation, but was not cytotoxic to normal hematopoietic cells or stem cells 2, 16. Therefore, due to the preclinical efficacy of tigecycline in AML cells as well as its safety as an antimicrobial, we carried out a phase I dose‐escalation study of intravenous tigecycline in patients with relapsed and refractory AML.

## Methods

### Patient eligibility

Patients at least 18 years of age with relapsed or refractory AML were eligible to participate in this study. Eligible patients included those with no other potentially curative or standard salvage therapy options, or without prior treatment who are not eligible for induction chemotherapy as defined by age ≥80 or age >70 with poor risk cytogenetics (3 or more abnormalities, ‐5/del(5q), 3q abnormalities, or ‐7), or stable comorbidities that would preclude induction chemotherapy such as left ventricular ejection fraction (LVEF) <40% and/or diffusing capacity of the lungs for carbon monoxide (DLCO) <60% expected.

Patients were required to have an Eastern Cooperative Oncology Group (ECOG) performance status of ≤2 and normal liver and kidney function (total serum bilirubin <1.5 times, serum creatinine <2 times, aspartate aminotransferase and alanine aminotransferase <2 times the upper limit of normal [ULN]). In addition, study entry required recovery (≤ grade 1) from nonhematologic toxicity from prior chemotherapy, and patients were required to provide informed written consent and comply with the study procedures to be eligible.

Patients were excluded from the study if they were allergic to tetracycline or minocycline, received tigecycline in the last month prior to registration, or at any point as an anticancer agent. In addition, patients receiving chemotherapy, other than hydroxyurea, to control circulating blast counts or receiving concomitant therapy with linezolid or chloramphenicol, compounds that are known to inhibit mitochondrial protein synthesis, were not eligible. Patients with uncontrolled medical illnesses, infections or psychiatric conditions, were excluded from the study. Patients using any other investigational antileukemic therapy within 14 days of registration of the study were ineligible. Women who were pregnant or breastfeeding were not considered for this study.

### Study drug

Tigecycline was purchased from the local pharmacy of each institution as a sterile, lyophilized orange powder or cake. Each glass vial contained 50 mg of tigecycline for intravenous use. Each vial was reconstituted as per the manufacturer's instructions (Pfizer, NY, NY) with 5.3 mL of 5% dextrose, 0.9% sodium chloride, or lactated Ringer's injection and swirled to dissolve the drug yielding a yellow to orange solution containing tigecycline at a concentration of 10 mg/mL. Before administration, reconstituted tigecycline was diluted by immediately withdrawing the drug from the vial and adding it to 500 mL of 0.9% sodium chloride. The maximum infusion concentration was 1 mg/mL.

Prior to reconstitution, tigecycline was stored at 20 to 25°C. Once reconstituted, tigecycline was allowed to be stored at room temperature for up to 24 h (up to 6 h in the vial and the remaining time in the intravenous bag). Alternatively, reconstituted tigecycline was stored refrigerated at 2–8°C for up to 48 h following immediate transfer of the reconstituted solution into the intravenous bag. The drug was infused over 1 h ± 10 min. Since tigecycline has a reported t_1/2_ of 27–43 h and was stable for only 6 h at room temperature in the vial, this prevented continuous infusion and once daily dose of tigecycline was selected for this study.

To prevent nausea and vomiting associated with tigecycline, patients were instructed to consume a meal 2 h prior to drug administration. In addition, patients received granisetron (2 mg orally or 1 mg intravenously) or a suitable alternative 30 min prior to tigecycline administration. Additional antiemetics were given at the discretion of the treating physician. Of note, consumption of food does not affect the tigecycline concentration in the serum 17.

The investigational new drug (IND) application for tigecycline was held by University Health Network and obtained using the product insert of tigecycline that provided information on drug safety, pharmacokinetics, and metabolism.

### Trial design

This was a phase I open‐label, multicenter, 3+3 dose‐escalation study of tigecycline in patients with AML. One cycle of treatment was defined as 3 weeks, of which tigecycline was administered intravenously daily for 5 of 7 days (Monday–Friday) for 2 weeks followed by a rest period of 1 week. Patients continued with treatment cycles until disease progression. Three patients were enrolled and treated at each dose level in sequential cohorts according to the schedule in Table 1. The cohort was expanded if 1 of the 3 patients experienced a dose‐limiting toxicity (DLT). In that case, three more patients were enrolled with the same dose. If none of these three additional patients experienced a DLT in cycle 1, dose escalation was allowed.

**Table 1:** Tigecycline dose escalation schedule

Dose level	Administered dose (mg)	*n*
1	50	3
2	100	3
3	150	3
4	200	5
5	250	4
6	300	4
7	350	5^a^
Total		27

One patient received cycle 1 at 350 mg/m^2^ and cycles 2 and 3 at 300 mg/m^2.^

Additional patients could be enrolled at any dose level as the trial was conducted at multiple sites and multiple patients could have been offered the trial simultaneously as enrollment was competitive. Patients were enrolled and started on therapy prior to the previous patient completing the full course of therapy. All adverse events were rapidly reported to closely oversee the progress of all patients on study and detect important adverse events that may have required modifying or stopping the study.

A DLT was defined as any grade 3, 4, or 5 adverse event attributable to study drug (definitely, probably, or possibly) during the first cycle of treatment. At the investigator's discretion, grade 3 nausea, or grade 3 or 4 vomiting, if manageable with supportive care measures, was not considered a DLT. Dose escalation was permitted if no DLTs were observed in the first three patients or no more than 1 DLT among six patients were encountered in the first cycle.

The maximally tolerated dose (MTD) was defined as the next lower dose below the one where DLTs precluding dose escalation were observed. If the MTD was not reached by dose level seven, no further dose escalation was allowed. Patients were withdrawn from treatment in the event of grade 3 or 4 toxicity occurring during cycle 1 of treatment. If grade 3 or 4 toxicity occurred after cycle 1 of treatment, therapy with tigecycline was stopped until toxicity was resolved to grade 1 or less, and the treatment reduced by one dose level.

### Endpoints and assessment

Patients that progressed during or after completing the 3‐week treatment cycle were withdrawn from the study. Patients that did not progress and did not have evidence of significant toxicity were eligible to receive additional cycles of tigecycline. The following response criteria was used: Complete response (CR): <5% blasts in a normocellular bone marrow, absolute neutrophil count (ANC) >1.0 × 10^9^/L and platelets >100 × 10^9^/L, and no extramedullary disease; Complete response, incomplete platelet recovery (CRp): meets the criteria for CR except platelets <100 × 10^9^/L, but independent of platelet transfusions; partial response (PR): at least 50% reduction in bone marrow blasts with 5–15% residual marrow blasts, ANC >1.0 × 10^9^/L and platelets >50 × 10^9^/L, independent of transfusions; morphologic leukemia‐free state (MLF): <5% blasts in an assessable sample with at least 200 nucleated cells counted without neutrophils and platelet recovery; and no response (NR): does not meet the criteria for CR, CRp, PR, or morphologic leukemia‐free state.

### Pharmacokinetics

Five mL of peripheral blood samples were acquired from patients enrolled in the study in heparinized tubes on screening day, at 1 h prior to infusion, at the end of infusion (0 h), and at 0.5, 1, 2, 4, and 24 h after the end of infusion. It should be noted that no end of infusion samples were acquired for the three patients at the 50 mg dose. Additional samples were acquired prior to infusion on days 3, 4, 5, and 12. Within an hour postcollection, blood samples were centrifuged at 1500*g* for 10 min at 4°C to collect plasma layer, which was frozen until analysis. Plasma concentrations of tigecycline were determined using a fully validated LC‐MS/MS method, and resulting values were used for noncompartmental analysis. Observed C_max_, areas under the curve (AUC) from 0–24 h is calculated using linear trapezoidal extrapolation, and t_1/2_ are reported.

### Pharmacodynamics

Peripheral blood (20 mL) was obtained pretreatment and during the cycle one on patients with circulating blasts. Mononuclear cells were isolated by Ficoll–Hypaque separation and depleted of CD3^+^, CD19^+^ and glycophorin A^+^ cells using one round of EasySep^™^ negative selection (StemCell Technologies, Catalog no.: 19309). Negative selection was omitted for patients 8, 11, 13, 19, 21, 23, and 24 due to low mononuclear cell count after Ficoll–Hypaque separation. Negative selection was performed according to manufacturer's protocol. Briefly, mononuclear cells (5 × 10^7^ cells/mL) were suspended in phosphate buffer saline (PBS) with 2% fetal bovine serum (FBS) and 1 mmol/L ethylenediaminetetraacetic acid (EDTA) and incubated with negative selection cocktail at room temperature for 10 min. Magnetic nanoparticles were added to the mix and the suspension incubated at room temperature for 10 min followed by separation.

120 *μ*L of nDBM lysis buffer (n dodecyl beta maltoside (Sigma, Catalog no.: D4641) made in PBS with protease inhibitors was added to 5 × 10^6^ cells and incubated on ice for 30 min with periodic vortexing. Samples were centrifuged at 15,800 × g for 20 min at 4°C, and the protein concentration of the cell lysate was measured, using the DC protein assay. Samples were not boiled after the addition of loading buffer to avoid protein precipitation 2, 18 and 20 *μ*g of protein was loaded per lane for gel electrophoresis. Levels of Cox‐1 and Cox‐4 protein were analyzed using immunoblotting. Anti‐Cox‐1 antibody (Santa Cruz Biotechnology Inc., Catalog no.: sc‐58347) and anti‐Cox‐4 antibody (Molecular Probes, Catalog no.: A21347) were used at 1:1000 and 1:5000 dilution, respectively, and incubated overnight at 4°C. Secondary antimouse HRP (GE Healthcare Life Sciences, catalog no.: NA931V) antibody was used at 1:1000 dilution and incubated for 1 h at room temperature. Quantitative densitometry was performed on the western blots to calculate the ratio of Cox‐1:Cox‐4 on different days. Samples from the following patients were excluded from the analysis due to lack of sufficient protein for immunoblotting (patient 3, 5, 6, 8, 13, 14, 17, and 19) or technical challenges with the assay (patients 4 and 9).

1x10^7^ mononuclear cells were used to isolate mRNA using the RNeasy mini kit (Qiagen, Catalog no.: 74134). Isolated mRNA was stored at −80°C and transferred to a facility for NanoString analysis. Cox‐1 mRNA was analyzed using the NanoString protocol (NanoString Technologies Inc.). Samples from patients 21, 23, 24, 25, and 27 were not analyzed for mRNA due to lack of sample amount.

### Statistical analysis

Statistical analyses were primarily descriptive in nature and a summary of statistics was carried out for all safety and efficacy parameters by dose group. For continuous variables, data was presented using number, mean, and median; standard deviation; and minimum and maximum. For categorical variables, summary tabulations of the counts and percentages in each parameter were presented.

## Results

### Demographics

The demographics of the patients enrolled in the study are outlined in Table 2. Twenty‐seven patients with AML were enrolled in the study from June 2011 to July 2014; all patients received tigecycline and were included in the analysis. The median age of patients was 70 years (range 44–84 years) and 59% were male. One patient (4%) had good‐risk cytogenetics, 12 patients (44%) had intermediate‐risk cytogenetics, seven patients (26%) had poor‐risk cytogenetics, and the karyotype was unknown in seven patients (26%) using previously established criteria for cytogenetic risk 19. Patients had a median of two prior therapies (range 0–8). A total of 42 cycles were administered over seven dose levels of which 30 were completed (71%) by 22 patients. A summary of the number of study cycles per patient at different dose levels is shown in Table 3. Ten patients received more than one cycle and one patient received four cycles of study drug.

**Table 2:** Demographics and baseline characteristics of patients enrolled in the study

	*n*
*n*	27
Age Median (range), years	70 (44–84)
Male	16
Diagnosis	
Relapsed AML	15
Refractory AML	10
AML; not eligible for chemotherapy	2
Cytogenetic Risk Group	
Good	1
Intermediate	12
Poor	7
Unknown	7
Prior therapies Median (range)	2 (0–8)
IC: Induction Chemotherapy	24
PC: Postremission Chemotherapy	8
HSCT: Hematopoietic Stem Cell Transplantation	2
Other	4
Total cycles administered	42
Total cycles completed	30

AML, Acute myeloid leukemia.

**Table 3:** Study drug exposure in patients treated with tigecycline

Dose (mg)	Total cycles per patient^a^, *n* (%)			
	1	2	3	4
50	1 (33)	2 (67)		
100	3 (100)			
150	2 (67)	1 (33)		
200	3 (60)		1 (20)	1 (20)
250	2 (50)	1 (25)	1 (25)	
300	3 (75)	1 (25)		
350	3 (60)	1 (20)	1 (20)^b^	
Total	17 (63)	6 (22)	3 (11)	1 (4)

a Cycles not completed have also been counted.

b Cycle 1 at 350 mg/m^2^ and cycles 2 and 3 at 300 mg/m^2.^

### Safety

No grade 3 or 4 toxicity related to study drug was observed up to 300 mg/day, the first six dose levels. Two patients experienced DLTs related to tigecycline at the 350 mg/day level (7th dose level). On the last day of cycle 1 at the 350 mg/day dose level, a 75‐year‐old female experienced an asymptomatic and reversible grade 3 elevated aspartate aminotransferase and grade 3 congestive heart failure that were judged to be related to tigecycline treatment. Prior to starting the study, this patient had risk factors for cardiomyopathy, fluid retention and mild edema of the legs. By MUGA scan, the ejection fraction was 57% prior to the study and decreased to 30% at the time of the DLT. The levels of aspartate aminotransferase decreased toward normal within a week after the patient was taken off tigecycline, but the heart failure remained unchanged until her death from progressive disease a month later.

Another patient in this dose cohort, a 78‐year‐old female, tolerated tigecycline well for the first 5 days, but developed an asymptomatic grade 3 increase in serum amylase and a grade 3 increase in lipase on day 8 that were possibly related to tigecycline. The drug was stopped and the abnormal enzyme levels returned to normal after 10 days off study drug. No other causes for pancreatitis, such as gall stones, were identified.

In addition, two patients in the 200 mg/day dose cohort were taken off treatment due to grade 3 or 4 AEs, which were judged to be unrelated to tigecycline treatment. A 70‐year‐old male experienced grade 3 hyperbilirubinemia after 5 days of treatment in cycle 1 and an 84‐year‐old male experienced grade 3 atrial fibrillation on day 9 of treatment in cycle 1. Also on day 10 of cycle 1, a 77‐year‐old male in the 350 mg/day cohort experienced a grade 3 elevated aspartate aminotransferase that was judged to be unrelated to tigecycline treatment. The patient was taken off treatment to resolve the elevated liver enzymes. Furthermore, a 69‐year‐old male in the 150 mg/day dose cohort died of grade 4 septic shock and neutropenic fever after completing cycle 1. The death was judged to be unrelated to tigecycline treatment.

The other AEs greater than grade 1 that were possibly related to tigecycline treatment were nausea, diarrhea, and elevated lactate dehydrogenase levels in two patients each, and elevated bilirubin levels, hypoalbuminemia, fatigue, vomiting, and anorexia in one patient each (Table 4). These AEs developed in patients at dose levels of 150 mg/day tigecycline and higher, while no AEs developed at the first two dose levels of 50 and 100 mg/day tigecycline. Thus, the MTD of tigecycline when given as a once daily 1 h infusion in this study was 300 mg/day.

**Table 4:** Summary of adverse events related to tigecycline by dose level and grade based on events

Dose (mg)	Adverse event	All grade	Grade ≥3
50	None observed		
100	Nausea	1	0
150	Hypomagnesemia	1	0
	LDH elevated	1	0
	Nausea	3	0
	Photosensitivity	1	0
	Vomiting	1	0
200	Blood bilirubin increased	1	0
	Diarrhea	1	0
	Fatigue	1	0
	Gastroesophageal reflex disease	1	0
	LDH elevated	1	0
	Nausea	3	0
	Pruritus	1	0
250	Diarrhea	2	0
	Fatigue	1	0
	Nausea	1	0
	Vomiting	2	0
300	Anorexia	1	0
	Diarrhea	1	0
	Hypomagnesemia	2	0
	Nausea	2	0
	Vomiting	1	0
350	Alanine aminotransferase elevated	1	0
	Alkaline phosphatase elevated	1	0
	Aspartate aminotransferase elevated	1	1
	Diaphoresis	1	0
	Diarrhea	1	0
	Heart failure	1	1
	Hot flashes	1	0
	Hypoalbuminemia	1	0
	Lipase elevated	1	1
	Nausea	3	0
	Serum amylase elevated	1	1
	Vomiting	1	0
	Weight gain	2	0

### Disease response

None of the patients had evidence of disease response. The treatment was terminated because patients had progressive disease or no response (18 patients, 67%), grade 3 or 4 AEs (three patients, 11%), AEs unrelated to tigecycline (two patients, 7%), withdrawn from treatment (three patients, 11%), or died (one patient, 4%).

### Pharmacokinetics

**Figure 1:**
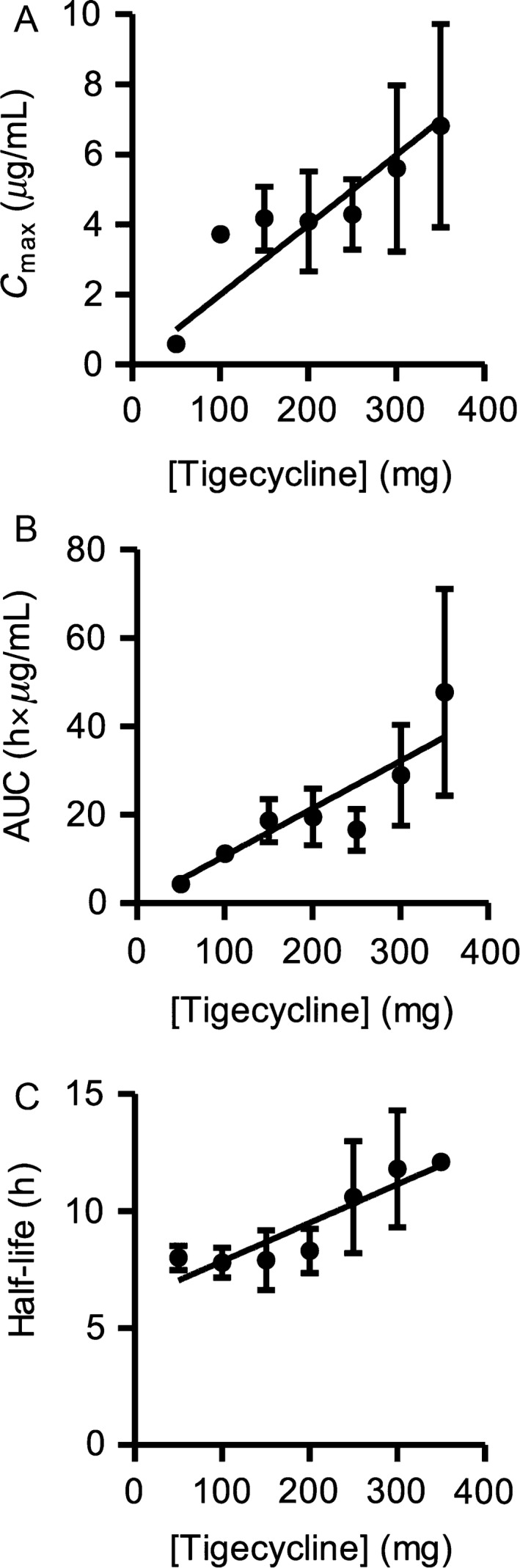
Plasma levels of tigecycline are proportional to the administered dose. (A) The peak plasma concentrations (C_max_), (B) areas under the curve (AUC) through 0–24 h, and (C) half‐life following administration of tigecycline. Peripheral blood samples were collected at predose (−1 h), and 0, 0.5, 1, 2, 4, and 24 h postdose. End of infusion samples were not acquired for the patients at the 50 mg dose. Additional predose samples were collected on days 3, 4, 5, and 12. Plasma concentrations of tigecycline were determined using a fully‐validated LC‐MS/MS method, and resulting values were used for noncompartmental analysis. Data are presented as mean ± SEM.

Pharmacokinetic data was obtained from all 27 patients and T_max_ was observed at the end of infusion. Since samples were not acquired until 30 min past the end of infusion for the three patients at the 50 mg dose level, the observed C_max_ values are below the true C_max_ at that dose. As shown in Figure 1A and B, C_max_ and AUC increased as a function of dose and appeared linear throughout the 0–350 mg dose range.

Semilogarithmic plots of plasma concentration versus time exhibited a constant slope between 2 and 24 h, supporting the determination of an apparent half‐life (t_1/2_). The apparent t_1/2_ of tigecycline appeared to increase slightly as a function of dose, and had a mean value of 9.5 ± 1.9 h and a range of 6.0–14.6 h over the dose range (Fig. 1C). This half‐life is shorter than published half‐lives for tigecycline that range from 27 to 43 h 17, 20. Based on the published half‐life values of tigecycline, steady‐state should not have been achieved until at least day 5, and based on the ratio of half‐life to dosing interval that steady‐state trough concentration should have increased markedly following these daily infusions. Instead, consistent with the relatively short t_1/2_ determined in this study, the measured predose tigecycline concentrations on days 2–12 did not show a significant or consistent increase above the day two values (Figure S1). The apparent increase in predose concentration seen on day 4 for the 350 mg dose cohort is skewed by a very high measured value for one patient, and that increased predose concentration is neither maintained nor increased further on subsequent days. This supports our conclusion that the predose concentration at day four is an outlier, and not indicative of true accumulation of drug.

### Pharmacodynamics

**Figure 2:**
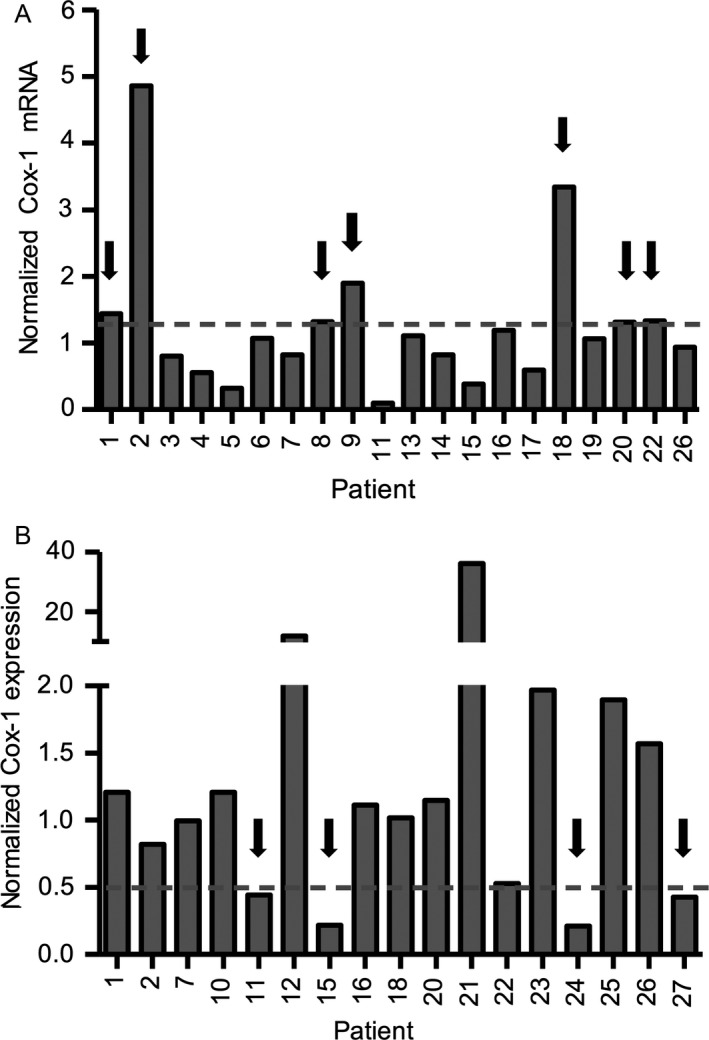
Pharmacodynamic changes in mitochondrial proteins after treatment with tigecycline. Leukemic blasts from peripheral blood samples were obtained on screening day, predose and on days 4, 8 and 11/12 postdose (−1 h) after tigecycline treatment. Mononuclear cells were isolated, using Ficoll–Hypaque separation followed by EasySep^™^ negative selection, other than samples from patients 8, 11, 13, 19, 21, 23, and 24 for which negative selection step was omitted. (A) Cox‐1 and *β*2M were measured by NanoString protocol in mononuclear cells from patients before and after treatment with tigecycline. Samples before tigecycline treatment were predose from day 1 or screening day samples for patients 6 and 14. Samples after tigecycline treatment were from day 11/12 (or on day 8 for patients 8, 15, 17, 22 and 26 or on day 5 for patient 13). Patients 10, 12, and 20 were excluded due to missing pretreatment samples, and mRNA analysis was not carried out for patients 21, 23, 24, 25 and 27. Cox‐1 RNA levels were normalized to *β*2M levels. Values of 1 indicate no change, <1 indicate a decrease, and >1 indicate an increase in Cox‐1 RNA levels due to tigecycline treatment. Dotted line indicates an arbitrary cut off at 1.3 times increase from control. **(B)** Expression of Cox‐1 and Cox‐4 proteins was measured by immunoblotting and analyzed using densitometry. To calculate a change in Cox‐1 levels relative to Cox‐4 levels, the ratio of Cox‐1 to Cox‐4 from day 11/12 was divided by that from the predose day. Day 11/12 samples were not available for some patients, therefore day 10 samples were used for patients 18 and 23, and day 8 samples were used for patients 10, 12, 15, 20, 22, and 26. In addition, predose samples were not available for patients 7, 11 and 12, therefore, samples from screening day were used. Values of 1 indicate no change, <1 indicate a decrease, and >1 indicate an increase in Cox‐1 levels due to tigecycline treatment. Dotted line indicates an arbitrary cut off at 2 times decrease from control.

We previously showed that tigecycline inhibits mitochondrial protein synthesis in AML cells resulting in a reduction in mitochondrially encoded proteins such as Cox‐1 but no change in nuclear encoded proteins such Cox‐4 that are both components of respiratory chain complex IV 2, 8. Along with the reduction in mitochondrially encoded proteins, we observed a concomitant and compensatory upregulation of mRNA encoding the mitochondrial proteins. Peripheral blood was obtained from all 27 patients pre and posttreatment with tigecycline. Mononuclear cells were isolated and changes in mRNA and mitochondrial proteins were measured by NanoString RNA analysis and immunoblotting, respectively. Adequate material was available for mRNA analysis from 20 patients and for protein analysis from 17 patients. Consistent with the lack of clinical response and short half‐life of the drug, no significant pharmacodynamics changes were observed. We observed greater than a 1.3‐fold increase in the level of Cox‐1 mRNA compared to pretreatment levels in seven patients (patient 1 and 2 = 50 mg, patient 8 and 9 = 150 mg, patient 18 = 250 mg, patient 20 = 300 mg, and patient 22 = 350 mg) (Fig. 2A). Four patients had more than a twofold reduction in protein levels of Cox‐1 relative to Cox‐4 after treatment with tigecycline (patient 11 = 200 mg, patient 15 = 250 mg, patient 27 = 300 mg, and patient 24 = 350 mg) (Fig. 2B). However, there was no correlation between patients with increased Cox‐1 mRNA and decreased Cox‐1 protein levels.

## Discussion

We previously showed that mitochondrial biogenesis and energetics are dysregulated in AML cells, and pharmacologically targeting the mitochondrial protein translation is a novel antileukemic strategy 2, 5, 21. We identified tigecycline, an antimicrobial agent, from a chemical screen with anticancer activity in preclinical studies of AML 2. Tigecycline was preferentially cytotoxic to AML cells and leukemic stem and progenitor cells, compared to normal hematopoietic cells in vitro and in vivo 2. Motivated by these results, we conducted a phase I study of escalating doses of tigecycline in patients with relapsed and refractory AML. However, none of the patients had a clinical response, and the study was stopped after administering tigecycline to 27 patients for 42 cycles over seven doses.

In our study, the maximal evaluated dose was 350 mg/day and the maximal tolerated dose was 300 mg/day. Dose‐limiting toxicity was observed at 350 mg/day in two patients with adverse events including elevated aspartate aminotransferase and heart failure in one patient, and elevated serum amylase and lipase in the second patient. Increases in transaminases and pancreatic enzymes, and congestive heart failure have been reported in patients treated with tigecycline and are listed as rare drug reactions in the tigecycline package insert 22, 23. In addition, nausea and vomiting are the most frequently reported adverse events for the use of tigecycline 17. In this study, grade‐2 nausea, diarrhea and vomiting were reported in a total of five patients and judged to be possibly related to the treatment.

In our study, tigecycline had a t_1/2_ of about 9.5 h which is 3–4.5‐fold lower than previously reported t_1/2_ values 17, 20, 24, 25. Tigecycline is not extensively metabolized and is primarily cleared through biliary excretion (59%) and renal elimination (32%) 26, 27. The tigecycline C_max_ values obtained following 300 mg/day were 12 *μ*mol/L, which is sufficient concentration to reduce the viability of AML cell lines and primary leukemic patient samples 2, 16. However, at serum concentrations higher than ~2 *μ*mol/L tigecycline, the protein binding for tigecycline can exceed 87% 25. Therefore, this is a possible explanation for not observing pharmacodynamics or clinical changes despite achieving concentrations that induced cell death in culture. In addition, the measured predose concentrations on days 2–12 of cycle 1 indicate that the steady‐state levels of tigecycline were ~1 *μ*mol/L at the 300 mg/day dose, a concentration at which we did not observe any in vitro antileukemic activity 2, 16. If intracellular and particularly intramitochondrial tigecycline concentrations follow the plasma concentration time course, then it is likely that there will be significant intervals between infusions when the concentration of the drug is below its effective range.

Our C_max_ values are from 1.5 to 4 times higher than previously reported values at the same doses, and all AUC calculated values were approximately twofold higher than the reported values 17. Repeat analysis of plasma aliquots from several patients resulted in approximately the same concentrations. Moreover, our calibration standards and quality control samples consistently fell within range, supporting the validity of our measurements. Two possible explanations are proposed for this quantitative difference between our data and those reported by Muralidharan et al. 17. The first is based on the analytical technique: the improved sensitivity and specificity of the current UPLC‐MS/MS analytical method relative to those obtained with a typical HPLC‐UV‐based method may provide a more accurate measurement of true tigecycline concentrations. The second possible explanation is biological: the pharmacokinetics of tigecycline in these cancer patients may be quantitatively different than the pharmacokinetics of the drug in the healthy subjects recruited for the published study.

We have previously shown that tigecycline inhibits mitochondrial protein translation in AML cells. Therefore, we conducted pharmacodynamic analyses during this study to evaluate the effects of tigecycline on the mitochondrial protein translation by measuring the levels of mitochondrially translated protein, Cox‐1, and comparing it to the nuclear translated protein, Cox‐4. No significant pharmacodynamic responses were observed, consistent with the lack of clinical response. Potentially, greater target inhibition might have been noted if a steady‐state concentration of greater than 1 *μ*mol/L of tigecycline could have been achieved.

As an antimicrobial, tigecycline reversibly binds to the 30S subunit of the bacterial ribosome, blocking the aminoacyl‐tRNA from entering the A site, thereby inhibiting elongation of the peptide chain and protein synthesis in bacteria 13, 15. Therefore, it is expected that tigecycline also binds to the mitochondrial ribosomes as a reversible inhibitor. One reason for the lack of response in this trial was that the drug was not on‐target for a sufficient time to inhibit mitochondrial protein translation.

A continuous intravenous infusion of tigecycline could be explored as a strategy in future studies to achieve a higher steady state concentration. However, the approved formulation of tigecycline is relatively unstable after reconstitution, making continuous infusion of the drug challenging for ambulatory patients. To increase the stability of tigecycline after reconstitution and permit the evaluation of continuous infusions of the drug, we have identified a new formulation of tigecycline containing ascorbic acid and vitamin E that maintains the stability of tigecycline for at least 7 days 16.

Antibiotics that inhibit bacterial protein synthesis have been reported to cross‐react with human mitochondrial ribosomes and inhibit mitochondrial protein synthesis 28. For example, chloramphenicol can cause bone marrow suppression, which has been attributed to inhibition of mitochondrial protein synthesis by binding the A‐site of the mitochondrial ribosome 28. Oxazolidinones also can cause myelosuppression and inhibit human mitochondrial ribosomes 29, 30. Thus, while structurally distinct from bacterial and cytosolic ribosomes, antimicrobials that inhibit bacterial ribosomes have a tendency to cross react with mitochondrial ribosomes. However, linezolid and chloramphenicol are less potent inhibitors of mitochondrial protein synthesis than tigecycline, so they would likely be less effective as therapeutic agents for leukemia.

In conclusion, this study represents the first clinical evaluation of tigecycline as a single agent in relapsed and refractory AML patients. The maximal evaluated dose was 350 mg/day and the maximal tolerated dose was 300 mg/day. Dose‐limiting toxicity was observed at the highest dose level, but tigecycline showed a favorable safety profile at doses 300 mg/day and under. Pharmacokinetic studies indicate that daily one‐hour infusions of tigecycline did not achieve and maintain effective steady‐state levels due to a short t_1/2_. Consistent with this finding, no clinical or pharmacodynamic responses were seen. Future studies should utilize the more stable formulation of tigecycline to permit continuous infusion to increase the concentration of tigecycline in the leukemic cells. Potentially through this approach, greater biological and clinical efficacy of tigecycline could be observed in patients with AML.

## Conflict of Interest

ADS and YJ hold equity in Trillium Therapeutics, and ADS is a member of their scientific advisory board.

## Supporting information

Figure S1. Predose concentrations of tigecycline by dose.
