# Supplementary material for: A Phase 1 study of intravenous infusions of tigecycline in patients with acute myeloid leukemia
Source: Cancer Med. 2016 Oct 13;5(11):3031–40. doi: 10.1002/cam4.845 (PMC5119957; doi:10.1002/cam4.845)
Supplement: Supplementary file 1 — Figure S1. Predose concentrations of tigecycline by dose. [file CAM4-5-3031-s001.doc]

**
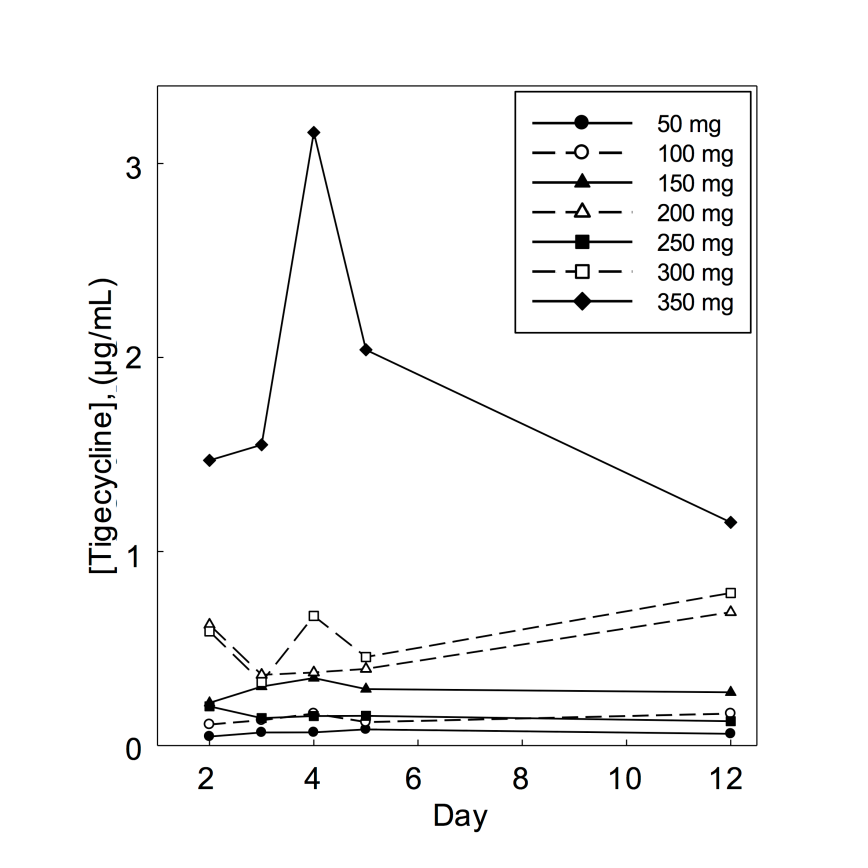
**

**Supplemental Figure 1: Pre-dose concentrations of tigecycline by dose.**

Mean pre-dose plasma tigecycline concentrations are presented for each dose. Peripheral blood samples were collected on the indicated days of cycle 1 of tigecycline treatment. Data are presented as mean concentrations for all included patients at a given dose level. Error bars are omitted for clarity.
